# Neurodevelopmental trajectories in well-controlled gestational diabetes mellitus offspring: No differences were found at the 6- and 12-month assessments

**DOI:** 10.3389/fendo.2025.1624334

**Published:** 2025-09-10

**Authors:** Jing Peng, Huazhang Miao, Li Zhang, Jing Jin, Lirong He, Dongdong Xue, Yong Guo, Guocheng Liu

**Affiliations:** ^1^ Department of Obstetrics, Guangdong Women and Children Hospital, Guangzhou, China; ^2^ Department of Health Care, Guangdong Women and Children Hospital, Guangzhou, China

**Keywords:** gestational diabetes mellitus (GDM), development, neurodevelopment, infant, blood glucose control

## Abstract

**Objective:**

To examine the associations between well-controlled gestational diabetes mellitus (GDM) and early neurodevelopmental trajectories in offspring.

**Methods:**

This retrospective cohort study included 2810 mother–infant pairs from Guangdong Women and Children Hospital (2016–2022). GDM was diagnosed via a 75 g oral glucose tolerance test at 24–28 gestational weeks, and women with well-controlled GDM were those who maintained blood glucose levels defined as a third-trimester HbA1c < 6% without requiring medication. Neurodevelopment was assessed via the Children’s Neuropsychological and Behavioral Scale-Revision 2016 at 6 and 12 months of age.

**Results:**

Among 2810 mother–infant pairs, 451 (16.05%) were diagnosed with GDM. Compared with non-GDM mothers, mothers with GDM had a greater median age (31.00 *vs*. 29.00 years; *P* < 0.001) and prepregnancy BMI (21.26 *vs*. 20.20 kg/m²; *P* < 0.001). No significant differences were observed in neonatal sex, birth weight or low birth weight (<2500 g) proportions. Neurodevelopmental assessments at 6 and 12 months revealed no significant differences in gross motor, fine motor, or adaptive behavior; language; or personal–social scores (all *P* > 0.05). Adjusted multivariate analyses revealed no associations between GDM and neurodevelopmental delay (≥2 subdomains below the threshold) at 6 months (OR = 0.92, 95% CI: 0.57–1.48; *P* = 0.739) or 12 months (OR = 0.87, 95% CI: 0.58–1.29; *P* = 0.479).

**Conclusions:**

Well-controlled GDM was not associated with adverse neurodevelopmental outcomes in early infancy, suggesting that optimized perinatal management may mitigate risks.

## Introduction

Gestational diabetes mellitus (GDM), the most prevalent metabolic disorder during pregnancy globally, affects 14.8% of pregnancies in mainland China (global range: 7.1%-27.6%) (1, 2). Its impact on maternal and neonatal health has expanded beyond traditional perinatal complications to encompass long-term neurodevelopmental effects in offspring (3, 4). Animal studies and epidemiological evidence suggest that GDM may disrupt fetal neurodevelopment through mechanisms involving intrauterine oxidative stress and chronic low-grade inflammation (5). Maternal hyperglycemia increases the production of reactive oxygen species, primarily via mitochondrial pathways, leading to membrane damage, activation of pro-apoptotic proteins, and excessive apoptosis, which can result in abnormal development of the fetal central nervous system (6). In addition, maternal hyperglycemia may elevate levels of pro-inflammatory cytokines, contributing to responses that could further impair fetal neurodevelopment, and potentially increase the risk of cognitive impairment, attention-deficit/hyperactivity disorder (ADHD), autism spectrum disorder (ASD), and motor dysfunction (5–10). A meta-analysis encompassing 56 million mother–child pairs (202 observational studies) confirmed significant positive associations between maternal diabetes and offspring neurodevelopmental disorders, with particular correlations for ASD and ADHD (11). These findings underscore the critical importance of early detection and management of maternal diabetes to improve childhood health outcomes.

However, the dynamic trajectory of GDM-related neurodevelopmental impacts remains controversial (12). Longitudinal cohort studies have demonstrated that male offspring of mothers with GDM exhibit persistent neurodevelopmental delays, particularly in problem-solving abilities, fine motor coordination, and personal–social functioning, with these deficits being detectable as early as 6 months and remaining clinically significant through 4 years of age (13). Critical modifiers include exposure duration, diagnostic timing, and disease severity: early diagnosis (≤26 weeks) correlates with elevated ASD/ADHD risk versus late diagnosis (>26 weeks), and pharmacologically managed GDM confers greater neurodevelopmental risk than does diet-controlled cases (11). Current clinical protocols enable glycemic target attainment in >80% of gestational diabetes cases via structured dietary interventions (carbohydrate-controlled meal plans with regulated meal timing), leading to marked reductions in obstetric complications, including fetal macrosomia and neonatal glucose dysregulation (14, 15). Nevertheless, robust evidence of the neurodevelopmental benefits associated with optimized maternal glycemic regulation in modern care paradigms is lacking. This retrospective cohort study systematically evaluated neurodevelopmental trajectories (motor, language, and social domains) in infants of mothers with well-controlled GDM, aiming to clarify neurodevelopmental patterns under current management paradigms and optimize perinatal interventions.

## Methods

### Study population

A retrospective cohort study involving pregnant women who received antenatal care and delivered at our facility between 2016 and 2022 was conducted at Guangdong Women and Children Hospital. The study protocol involved two phases of data collection: initial extraction of GDM screening records from electronic medical databases for women undergoing 75 g oral glucose tolerance test (OGTT) at 24–28 weeks gestation, followed by comprehensive manual review of complete medical records by investigators to verify data accuracy, including infant neurodevelopmental assessments at 6 and 12 months postpartum. The eligibility criterion was singleton pregnancies with term deliveries (≥37 weeks gestation). The exclusion criteria included multiple gestations, fetal demise, pregestational diabetes (type 1 or 2), insulin therapy, maternal chronic conditions (chronic hypertension or gestational hypertensive disorders, cardiopulmonary/hepatic/renal diseases), fetal chromosomal abnormalities, congenital malformations, and cases with incomplete documentation of critical information (OGTT results, neurodevelopmental evaluation outcomes, or essential pregnancy parameters).

### Ethical approval

The study protocol received ethical clearance from the Institutional Review Board of Guangdong Women and Children Hospital (Approval ID: 202201203). As this retrospective analysis utilized anonymized clinical data extracted from the institution’s electronic health records system, the ethics committee formally exempted the requirement for informed consent in compliance with national regulations governing deidentified medical data research.

### GDM assessment

The 75-g OGTT was administered to pregnant individuals between 24 and 28 gestational weeks. Diagnostic thresholds were defined as fasting glucose ≥5.1 mmol/L, 1-hour postload glucose ≥10.0 mmol/L, or 2-hour postload glucose ≥8.5 mmol/L. GDM diagnosis requires a single abnormal value meeting or exceeding these cutoffs (16). Third-trimester HbA1c measurements were obtained to evaluate glycemic management. Women with well-controlled GDM refers specifically to pregnant women who maintained good glycemic control throughout pregnancy (as defined by third-trimester HbA1c < 6% and no need for medication). In our retrospective cohort, only a very small proportion (about 1.1%, n=5) of GDM cases had poor glycemic control (third-trimester HbA1c > 6% or required pharmacological intervention), and these cases were excluded from our analysis.

### Neurodevelopmental assessment

The Children’s Neuropsychological and Behavioral Scale-Revision 2016 (CNBS-R2016) was employed to assess neurodevelopmental progression in infants at 6 and 12 months of age. This psychometrically validated instrument, developed through standardized protocols by the Capital Institute of Pediatrics (China), provides age-normed developmental benchmarks for children from 1 month through 72 months (17). As a clinician-administered diagnostic tool, it quantifies neuropsychological maturation through a composite developmental quotient (DQ) and five functional subdomains: gross motor, fine motor, language, personal-social, and adaptive behaviors. DQs are calculated via the following established formula: DQ = (Mental Age [months]/Chronological Age [months]) × 100. These metrics yield both domain-specific and composite scores, with clinical interpretation thresholds defined as follows: scores ≥80 reflect age-appropriate development, scores between 70–79 indicate mild developmental delay, and scores <70 signify clinically significant developmental delay.

### Statistical analysis

Analyses were performed via SAS 9.4 (SAS Institute) and R version 4.4.3 (R Core Team). The figures were plotted via R version 4.5.0 (R Core Team) and the ggplot2 package. Maternal and neonatal characteristics are presented as mean with standard deviation (SD) for continuous variables and as numbers (%) for categorical variables. Independent samples Satterthwaite t tests were conducted to examine differences in the means of continuous variables. Chi-square tests were used to compare differences in the proportions of categorical variables. The development delay rates with 95% CIs of the non-GDM and GDM groups at 6 months and 12 months were calculated via a binomial distribution exact method. Adjusted associations between development delay (≥2 subdomains) and GDM at 6 months and 12 months were examined separately via a multivariate logistic regression model adjusted for maternal age, BMI at conception, parity, delivery mode, infant sex, and birthweight. The LS mean and LS mean differences between the Non-GDM and GDM groups in terms of gross motor, fine motor, language, personal-social, and adaptive behaviors at 6 months and 12 months were calculated via a mixed model and adjusted for maternal age; BMI at conception; parity; delivery mode; infant sex; and birthweight. The confidence level was set at 95%, and *P <*0.05 was considered statistically significant.

## Results

This study included 2810 mother–infant pairs, 451 (16.05%) of which were diagnosed with GDM. As shown in **Table 1**, GDM mothers were more likely to be older, have a higher prepregnancy BMI, and have lower educational attainment. The GDM group had a significantly greater median maternal age (31.00 *vs*. 29.00 years; *P* < 0.001) and a greater proportion of women aged ≥35 years (19.07% *vs*. 11.02%). The proportion of college-educated individuals was significantly lower in the GDM group (66.17% *vs*. 75.67%; *P* = 0.023). The median prepregnancy BMI was greater in the GDM group (21.26 *vs*. 20.20 kg/m²; *P* < 0.001), with a significantly increased proportion of women with a BMI ≥24.0 (18.84% *vs*. 11.20%; *P* < 0.001). Nulliparity was less common (67.85% *vs*. 73.38%; *P* = 0.016), and cesarean delivery rates were higher in the GDM group (36.81% *vs*. 34.55%; *P* = 0.046). No significant differences were observed in neonatal sex, birth weight or low birth weight (<2500 g) proportions.

**Table 1:** Maternal and neonatal characteristics stratified by GDM status.

Covariates	Non-GDM (N = 2359)	GDM (N = 451)	Overall (N = 2810)	*P* value
Maternal age (years)				
Median (IQR)	29.00 (27.00-32.00)	31.00 (28.00-34.00)	29.00 (27.00-32.00)	<0.001
<25 years	219 (9.28)	17 (3.77)	236 (8.40)	<0.001
25–34 years	1880 (79.69)	348 (77.16)	2228 (79.29)	
≥35 years	260 (11.02)	86 (19.07)	346 (12.31)	
Maternal education				
College or higher	1785(75.67)	358(66.17)	2143(76.26)	0.023
High school	342(14.50)	60(11.09)	402(14.31)	
Junior high school or below	212(8.99)	29(5.36)	241(8.58)	
Missing	20 (0.85)	4 (0.89)	24 (0.85)	
Prepregnancy BMI (kg/m^2^)				
Median (IQR)	20.20 (18.60-22.07)	21.26 (19.52-23.24)	20.40 (18.75-22.31)	<0.001
<18.5	563 (23.87)	67 (14.86)	630 (22.42)	<0.001
18.5-23.9	1532 (64.94)	299 (66.30)	1831 (65.16)	
24.0-27.9	220 (9.33)	71 (15.74)	291 (10.36)	
≥28.0	44 (1.87)	14 (3.10)	58 (2.06)	
Parity				
Nulliparous	1731 (73.38)	306 (67.85)	2037 (72.49)	0.016
Multiparous	628(26.62)	145(32.15)	773(27.51)	
Delivery mode				
Vaginal delivery	1544(65.45)	284(62.97)	1828(65.05)	0.046
Cesarean section	815 (34.55)	166 (36.81)	981 (34.91)	
Missing	0 (0.00)	1 (0.22)	1 (0.04)	
Neonatal sex				
Female	1068 (45.27)	194 (43.02)	1262 (44.91)	0.406
Male	1291 (54.73)	257 (56.98)	1548 (55.09)	
Gestational age at birth (weeks)				
Median (IQR)	39.00 (38.00-40.00)	39.00 (38.00-39.00)	39.00 (38.00-40.00)	<0.001
Birth weight (kg)				
Median (IQR)	3.21 (2.97-3.48)	3.20 (2.93-3.44)	3.21 (2.96-3.47)	0.105
<2500 g	67 (2.84)	15 (3.33)	82 (2.92)	0.589
2500–3999 g	2222 (94.19)	426 (94.46)	2648 (94.23)	
≥4000 g	70 (2.97)	10 (2.22)	80 (2.85)	
OGTT-0 h (mmol/L)				
Median (IQR)	4.30 (4.12-4.51)	4.56 (4.31-4.92)	4.33 (4.14-4.57)	<0.001
OGTT-1 h (mmol/L)				
Median (IQR)	7.48 (6.50-8.37)	10.09 (9.34-10.84)	7.80 (6.74-8.95)	<0.001
OGTT-2 h (mmol/L)				
Median (IQR)	6.41 (5.71-7.17)	8.95 (8.50-9.64)	6.70 (5.88-7.69)	<0.001
HbA1c (%)				
Median (IQR)	5.00 (4.75-5.20)	5.20 (5.00-5.40)	5.00 (4.80-5.20)	<0.001

**Figure 1:**
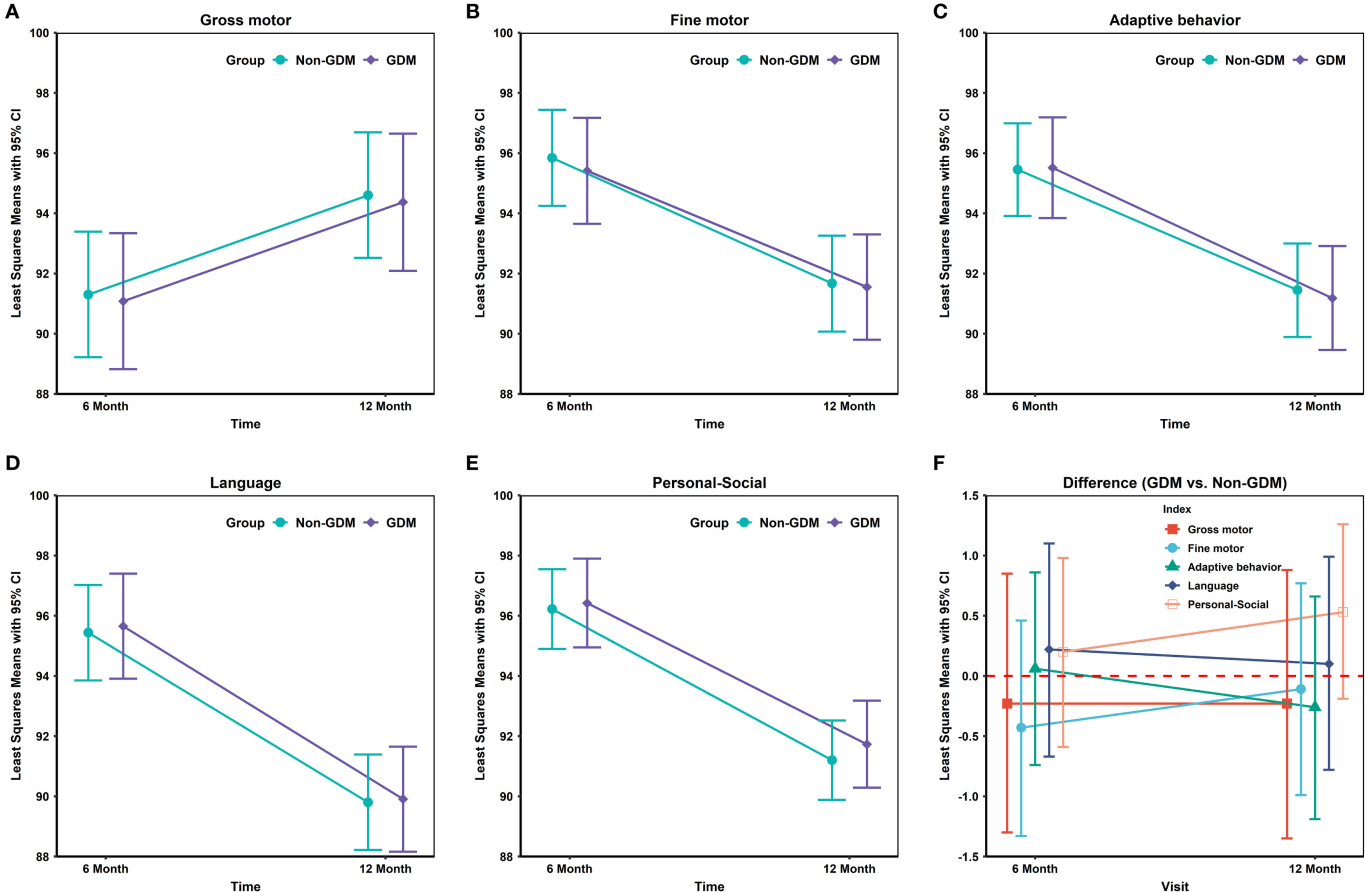
Differences in neurodevelopmental domain scores between GDM- and non-GDM-exposed infants at 6 and 12 months.

In **Table 2** and **Figure 1**, neurodevelopmental levels at 6 and 12 months were compared between infants of mothers with and without GDM. No statistically significant differences were observed in gross motor, fine motor, adaptive, language, or personal–social development scores (all *P* > 0.05). **Table 3** presents adjusted associations between GDM and neurodevelopmental delay (≥2 subdomains below the threshold) at 6 and 12 months of age, with odds ratios of 0.92 (95% CI: 0.57–1.48; *P* = 0.739) and 0.87 (0.58–1.29; *P* = 0.479), respectively, indicating no statistically significant associations.

**Table 2:** Neurodevelopmental levels in infants of mothers with GDM *vs* non-GDM mothers at 6 and 12 months.

Neurodevelopment	LS Mean of Non-GDM	LS Mean of GDM	Difference of LS Mean	*P*
Gross motor				
6 Months	91.30(89.22-93.39)	91.08(88.82-93.34)	-0.23(-1.30-0.85)	0.679
12 Months	94.60(92.52-96.69)	94.37(92.09-96.65)	-0.23(-1.35-0.88)	0.681
Fine motor				
6 Months	95.84(94.25-97.44)	95.41(93.65-97.17)	-0.43(-1.33-0.46)	0.344
12 Months	91.67(90.07-93.26)	91.55(89.80-93.30)	-0.11(-0.99-0.77)	0.798
Adaptive behavior				
6 Months	95.45(93.91-96.99)	95.51(93.84-97.19)	0.06(-0.74-0.86)	0.877
12 Months	91.45(89.89-93.00)	91.18(89.46-92.91)	-0.26(-1.19-0.66)	0.577
Language				
6 Months	95.44(93.85-97.02)	95.65(93.91-97.40)	0.22(-0.67-1.10)	0.633
12 Months	89.80(88.22-91.39)	89.91(88.16-91.65)	0.10(-0.78-0.99)	0.819
Personal-Social				
6 Months	96.22(94.9-97.55)	96.42(94.95-97.90)	0.20(-0.59-0.98)	0.620
12 Months	91.20(89.88-92.52)	91.73(90.29-93.18)	0.53(-0.19-1.26)	0.148

**Table 3:** Associations between GDM and neurodevelopmental delay (≥2 subdomains below threshold) at 6 and 12 months of age.

Time	Non-GDM			GDM			*OR (95% CI)*	*x^2^*	*P*
	*N*	*n*	*Rate (95% CI)*	*N*	*n*	*Rate (95% CI)*			
6 months									
	2359	117	4.96 (4.12-5.91)	451	21	4.66 (2.91-7.03)	0.92 (0.57-1.48)	0.11	0.739
12 months									
	2359	183	7.76 (6.71-8.91)	451	32	7.10 (4.90-9.87)	0.87 (0.58-1.29)	0.50	0.479

ORs were adjusted for maternal age, prepregnancy BMI, parity, delivery mode, infant sex, and birthweight.

## Discussion

This study evaluated neurodevelopmental outcomes at 6 and 12 months of age in the offspring of mothers with GDM and non-GDM controls. No significant differences were observed across five domains—gross motor, fine motor, language, personal-social, and adaptive behavior—suggesting that GDM has no clear adverse effect on early neurodevelopment. These findings align with those of several prior studies but contrast with those of most reports suggesting adverse neurodevelopmental effects of GDM.

Recent large-scale cohort studies and meta-analyses indicate a potential association between GDM and offspring neurodevelopmental abnormalities (11, 13). The underlying mechanisms may involve the chronic immune dysregulation characteristic of GDM, manifested through a cytokine imbalance and altered immune cell profiles that are similar to the immunological features observed in neurodevelopmental disorders (18, 19). Additionally, maternal hyperglycemia induces neonatal hypoglycemia, which may cause irreversible neurological damage if severe or prolonged (20). These early metabolic disturbances may subsequently contribute to impairments in multiple neurodevelopmental domains, including cognitive function, reading ability, and both gross and fine motor skills (21–23).

Research also provides conflicting evidence regarding GDM and offspring neurodevelopment. A Norwegian study reported no link between GDM risk and adverse neurodevelopmental outcomes (24), whereas an Indian study reported no reduction in cognitive ability among GDM offspring—with some scores even higher than those of controls (25). Our results partially support these observations, possibly because our cohort was derived from a single-center, well-managed prenatal care population. In current practice, most GDM patients receive standardized dietary guidance, weight management, and glucose monitoring, with only a minority requiring drug intervention. Systematic management may mitigate potential risks. The evidence suggests that optimal glycemic control during pregnancy normalizes neurodevelopmental outcomes in offspring (26, 27). Our study demonstrated that offspring of well-managed GDM mothers with controlled glucose levels exhibit no significant neurodevelopmental delays in infancy. These findings underscore the clinical importance of contemporary GDM screening and management strategies in preventing early neurodevelopmental impairment.

In this study, neurodevelopmental assessment was conducted using the CNBS-R2016, a clinician-administered instrument validated for children aged 1–72 months in the Chinese population. This scale offers several advantages, including comprehensive coverage of developmental domains and robust applicability to the Chinese pediatric population. However, it may underestimate subtle or higher-order cognitive deficits, is susceptible to inter-rater variability, and limits cross-study comparability with cohorts assessed by international tools such as the Bayley Scales of Infant Development or Griffiths Scales. In addition, although this retrospective cohort study evaluated neurodevelopmental trajectories at 6 and 12 months through multidomain assessments, several limitations should be considered. The sample size may be underpowered to detect small effect sizes, especially since most GDM pregnancies demonstrated adequate glycemic control and severity-stratified analyses were not performed. Furthermore, the absence of extended follow-up beyond infancy restricts the detection of late-onset neurodevelopmental abnormalities, such as executive dysfunction and learning difficulties, which often emerge at school age. Lastly, important confounding variables, including infant feeding practices (particularly breastfeeding duration) and family environmental factors, which are well-established determinants of neurodevelopment (28), were not systematically collected, limiting our ability to adjust for these covariates.

## Conclusions

On the basis of current evidence and our findings, GDM with prenatal care and glycemic control does not appear to pose significant risks for infant neurodevelopmental outcomes. These results support the effectiveness of contemporary GDM management, but long-term follow-up studies are warranted to comprehensively evaluate the potential long-term effects of GDM on offspring neurodevelopment.

## Data Availability

The raw data supporting the conclusions of this article will be made available by the authors, without undue reservation.
